# Long-Term Effects of Paced Heart Rate on Central Blood Pressure in Patients with Sinus Node Dysfunction and Hypertension: A Randomised Crossover Study

**DOI:** 10.3390/medicina62102032

**Published:** 2026-10-20

**Authors:** Tuuli Joonsalu, Silver Heinsar, Martin Serg, Mart Kals, Jüri Voitk, Mihkel Zilmer, Jaan Eha, Priit Kampus

**Affiliations:** 1 Department of Cardiology, Institute of Clinical Medicine, University of Tartu, L. Puusepa 8, 50406 Tartu, Estonia; tuuli.joonsalu@kliinikum.ee (T.J.); martin.serg@regionaalhaigla.ee (M.S.); jaan.eha@kliinikum.ee (J.E.); priit.kampus@ut.ee (P.K.); 2 Heart Clinic, Tartu University Hospital, L. Puusepa 8, 50406 Tartu, Estonia; 3 Centre of Cardiology, North Estonia Medical Centre, J. Sütiste Tee 19, 13419 Tallinn, Estonia; jyri.voitk@regionaalhaigla.ee; 4 Critical Care Research Group, Institute for Molecular Bioscience, The University of Queensland, 306 Carmody Road, Brisbane 4072, Australia; 5 Estonian Genome Center, Institute of Genomics, University of Tartu, Riia 23b/2, 51010 Tartu, Estonia; mart.kals@ut.ee; 6 Lifeyear, Valukoja 10, 11415 Tallinn, Estonia; 7 Department of Biochemistry, Institute of Biomedicine and Translational Medicine, Centre of Excellence for Genomics and Translational Medicine, University of Tartu, Ravila 19, 50411 Tartu, Estonia; mihkel.zilmer@ut.ee

**Keywords:** heart rate, blood pressure, hypertension, sinus node dysfunction, sick sinus syndrome, pacemaker, pulse wave analysis

## Abstract

*Background and Objectives*: Prior cardiac pacemaker studies examining the relationship between heart rate and central (aortic) blood pressure (BP) have predominantly used acute designs, limiting generalisability to long-term settings. The aim of this study was to assess the long-term effects of two clinically realistic pacing rate strategies on the central BP profile in permanently paced sinus node dysfunction (SND) patients in sinus rhythm and on antihypertensive medication. *Materials and Methods*: In this prospective, single-centre, randomised, single-blind, exploratory crossover clinical trial, the study participants completed two eight-week intervention periods (DDD 55 and 75 bpm) separated by a two-week washout. Office peripheral and central haemodynamics were measured at baseline and at the end of each period non-invasively with an oscillometric cuff-based device (SphygmoCor XCEL). *Results*: The study included 22 subjects (median age 76 (IQR 73–78) years, 16 women). At DDD 55 bpm, central systolic BP (primary outcome) was higher than at DDD 75 bpm (mean 127.8 ± 13.4 and 121.3 ± 10.6 mmHg, respectively, *p* = 0.04), whereas systolic BP amplification (peripheral systolic BP—central systolic BP) and pulse pressure (PP) amplification (peripheral PP/central PP) were lower (systolic BP amplification: mean 8.5 ± 3.2 and 10.1 ± 2.9 mmHg, respectively, *p* = 0.02; PP amplification: mean 1.2 ± 0.1 and 1.3 ± 0.1, respectively, *p* < 0.01). *Conclusions*: Long-term pacing at a moderately accelerated physiological rate may be associated with lower central systolic BP compared with pacing at a slightly bradycardic rate, in older permanently paced SND patients with hypertension.

## 1. Introduction

Chronically elevated resting heart rate (HR) has independently been associated with adverse health outcomes in observational studies in the general population [1] and, even more so, among patients with cardiovascular risk factors or established disease, including hypertension [2,3,4,5,6]. Yet the benefits of pharmacological HR lowering in hypertension appear context dependent [7]. The matter is nuanced by the fact that peripheral blood pressure (BP) is not the same as central BP [8]—the latter being the pressure to which the heart, brain, kidneys and large arteries are exposed [9]—and that HR exerts differing effects on each. HR is a physiological contributor to central BP and, along with age, an important determinant of its relationship to peripheral pressure [10]. In simplified terms, HR tends to correlate positively with peripheral BP but inversely with central BP [11,12].

Whether a lower cardiac pacing rate is associated with a less favourable central BP profile than a higher pacing rate in permanently paced sinus node dysfunction (SND) patients with hypertension in a long-term setting remains unknown. In an era of growing interest in device-based neuromodulation strategies to lower BP in people with pacemakers [13], this question is potentially relevant. Prior pacemaker studies examining the relationship between HR and central BP have predominantly used acute designs [14,15] and heterogeneous cohorts, limiting generalisability to permanently paced SND patients. To address these gaps, the aim of the current exploratory study was to assess the long-term effects of two clinically realistic pacing rate strategies on the non-invasively derived central BP profile in adult subjects with SND, a permanent dual-chamber cardiac pacemaker and concomitant primary arterial hypertension.

## 2. Materials and Methods

This was a prospective, single-centre, randomised, single-blind exploratory crossover clinical trial performed from 2020 to 2022. This study is reported in accordance with the CONSORT 2025 Statement for randomised trials, and the completed CONSORT checklist is provided as a Supplementary File.

### 2.1. Study Population

All participants were recruited from the pacemaker outpatient clinic of the North Estonia Medical Centre, Estonia.

The inclusion criteria were as follows: age 18–80 years; dual-chamber cardiac pacemaker implanted due to SND at least 3 months earlier; antihypertensive medication for primary arterial hypertension; and sinus rhythm confirmed by 12-lead electrocardiogram (ECG) and pacemaker interrogation.

Participants were excluded if their mean seated office peripheral BP was ≥140/90 mmHg, or if atrial pacing was <80% or ventricular pacing was >3% on interrogation since the previous follow-up. Participants with major comorbidities (e.g., prior acute coronary syndrome or heart failure with reduced ejection fraction) were also excluded. The full list of exclusion criteria is provided in the Supplementary Materials.

Potential participants were identified during routine pacemaker follow-up based on pacemaker interrogation and review of the electronic medical record. Those considered potentially eligible were informed about the study and invited to screening visits if willing to participate. As no formal screening log was maintained, the total numbers assessed for eligibility and excluded before randomisation are unavailable.

### 2.2. Ethics

The study protocol was approved by the Research Ethics Committee of the University of Tartu. All participants gave their written informed consent. The study was registered at ClinicalTrials.gov (Identifier: NCT04309175).

### 2.3. Study Protocol

**Figure 1:**
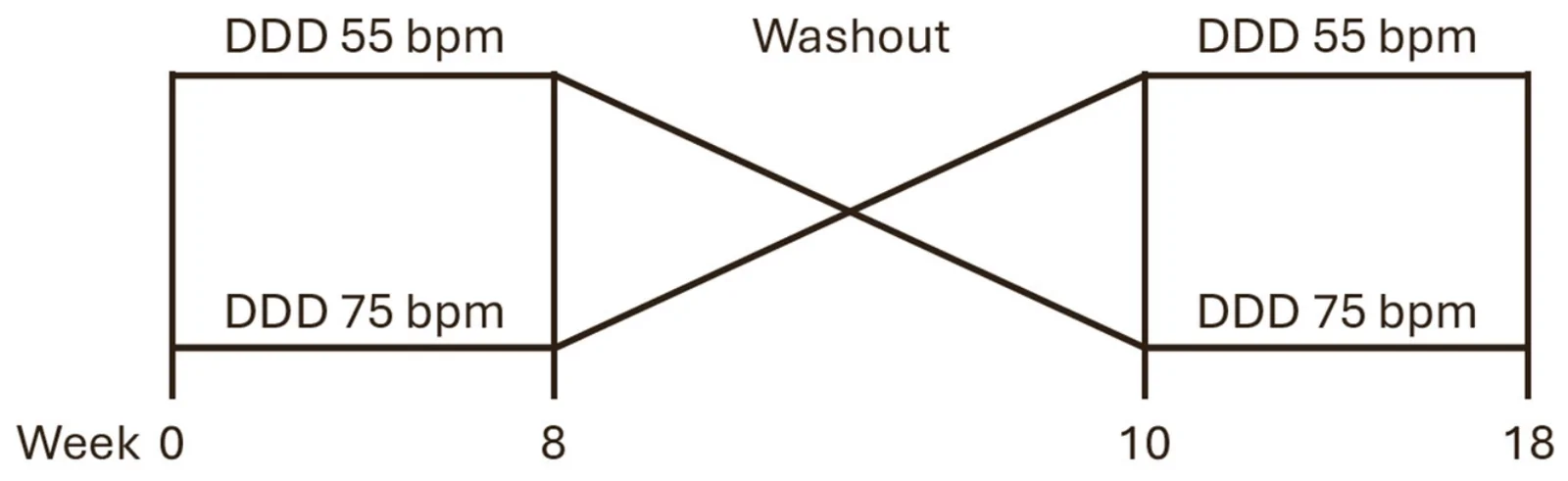
Study design and outline.

The study protocol (Figure 1) comprised two eight-week intervention periods separated by a two-week washout, and included two screening visits and four outpatient intervention visits. Intervention visits were conducted after an overnight fast with the participants continuing their daily cardiovascular medication during the study.

At the first intervention visit, baseline peripheral and central haemodynamics were recorded after at least five minutes of supine rest, after which the participants were allocated to one of the two pacing sequences, as described in detail below.

At week eight, clinical assessment, haemodynamic measurements, and pacemaker interrogation were repeated, and pre-intervention pacemaker settings were restored for washout. The second intervention period repeated the same procedures with participants crossed over to the alternate pacing setting.

Given the physiological nature of the primary outcome, the eight-week duration of each intervention period, and the design of the few previous non-acute crossover studies of cardiac pacing [16,17], a two-week washout period was considered appropriate to minimise potential residual haemodynamic effects after restoration of the pre-intervention pacemaker settings.

All measurements were performed under intracardiac electrogram monitoring, with each measurement obtained at least in duplicate and averaged using the two closest values.

### 2.4. Pacemaker Programming

All subjects had Abbott (formerly St. Jude Medical) pacemakers. Programming and continuous intracardiac electrogram monitoring were performed using the Merlin™ Patient Care System programmer (Abbott, Sylmar, CA, USA).

Permanent pacing was delivered in dual-chamber paced, dual-chamber sensed, dual response (DDD) mode at a lower rate limit of 55 bpm (lower pacing rate) or 75 bpm (higher pacing rate), with sensed/paced atrioventricular delays of 225/250 ms and ventricular intrinsic preference set to 100 ms to promote intrinsic atrioventricular conduction. Rate response and rate hysteresis were disabled.

### 2.5. Haemodynamic Measurements

Haemodynamic measurements were obtained non-invasively using the cuff-based oscillometric SphygmoCor XCEL system (AtCor Medical, Sydney, Australia) as described earlier [18]. Brachial pressure waveforms were recorded with an upper-arm cuff and used to derive the aortic waveform by pulse wave analysis (PWA). Carotid–femoral pulse wave velocity (cf-PWV) was assessed from simultaneous carotid tonometry and femoral cuff recordings as described earlier [19]. Path length was estimated using the subtraction method, and transit time was defined as the interval between the diastolic peaks of the carotid and femoral waveforms.

### 2.6. Laboratory Measurements

We performed blood sampling at screening. Laboratory measurements were done by standard methods in a local clinical laboratory.

### 2.7. Study Outcomes

The primary outcome of this exploratory study was central systolic BP. Secondary outcomes included peripheral BP (peripheral systolic and diastolic BP, peripheral pulse pressure (PP)), mean arterial pressure, central diastolic BP and central PP, central augmented pressure (AP), central augmentation index (AIx), PP amplification, systolic BP amplification, cf-PWV, P1 pressure and P1 height, P2 pressure and ejection duration.

### 2.8. Statistical Analysis

Baseline characteristics are reported as mean ± SD for normally distributed continuous variables, as median (IQR) otherwise, and as counts (%) for categorical variables. Because each participant was assessed at two pacing rate levels (DDD 55 bpm and DDD 75 bpm), associations between primary and secondary study outcomes and pacing rate were analysed using linear mixed-effects models with a random intercept for subject to account for the within-subject correlation. The models were adjusted for age. The models were fitted using the lmer function from the lme4 package in R. The statistical significance of fixed effects was evaluated using F-tests with the Kenward–Roger approximation for denominator degrees of freedom, as implemented in the lmerTest package. Estimated regression coefficients with 95% confidence intervals are reported. The proportion of variance explained by the models was quantified using conditional R^2^ calculated with the r.squaredGLMM function from the MuMIn package. To account for the crossover design, sensitivity analyses were performed in which period (first vs. second intervention period) and treatment sequence (DDD 55 → 75 bpm vs. DDD 75 → 55 bpm) were added as fixed effects to the linear mixed-effects models. In a two-treatment, two-period crossover design, the sequence effect is aliased with the carryover (treatment-by-period interaction) effect and was therefore used to assess potential carryover. Post hoc sensitivity analyses were performed for central and peripheral systolic BP by excluding participants who experienced atrial fibrillation episodes, excluding only measurements potentially affected by these episodes, and excluding each affected participant individually.

Secondary outcome analyses were considered exploratory, and no adjustment for multiple comparisons was applied. The association between the primary outcome—central systolic blood pressure—and pacing rate was additionally analysed after adjustment for peripheral systolic BP in a prespecified multivariable linear mixed-effects model. Spearman correlations were used to assess associations between central systolic BP (or central PP) and baseline cf-PWV. Outlier detection for baseline cf-PWV values was performed using Grubbs’ test using the outliers package. Subjects with missing data were excluded from multivariable analyses. All tests were two-sided and a *p*-value < 0.05 was considered statistically significant. All statistical analyses were performed using R version 4.3.1 (R Foundation for Statistical Computing, Vienna, Austria).

## 3. Results

### 3.1. Subjects

A total of 22 participants were randomised, with 10 allocated to the DDD 55 → 75 bpm sequence and 12 to the DDD 75 → 55 bpm sequence. No participants withdrew or were excluded after randomisation, and all 22 completed both intervention periods and were included in the analysis (Supplementary Figure S1). Their baseline characteristics are summarised in Table 1. In brief, 16 participants (73%) were female, and the median age of the subjects was 76 (IQR 73–78) years. Obesity (9/22, 41%), dyslipidaemia (18/22, 82%) and non-permanent atrial fibrillation or atrial flutter (16/22, 73%) were common comorbidities in the study population.

During the study, three participants experienced four atrial fibrillation episodes requiring rhythm restoration: one participant had two episodes during DDD 55 bpm, one had an episode during washout after DDD 55 bpm, and one had an episode during DDD 75 bpm. Two episodes were terminated with propafenone and two by electrical cardioversion. All haemodynamic measurements were performed during sinus rhythm.

### 3.2. Interventions

The mean paced HRs achieved at the lower and higher pacing rate levels were 54.5 ± 1.5 and 74.8 ± 1.4 bpm, respectively. The median percentage of atrial pacing was 89 (IQR 86–94)% at DDD 55 bpm compared with 99 (IQR 97–99)% at DDD 75 bpm. The median percentage of ventricular pacing was 1 (IQR 1–1)% at DDD 55 bpm and 1 (IQR 1–10)% at DDD 75 bpm.

### 3.3. Primary Outcome

**Figure 2:**
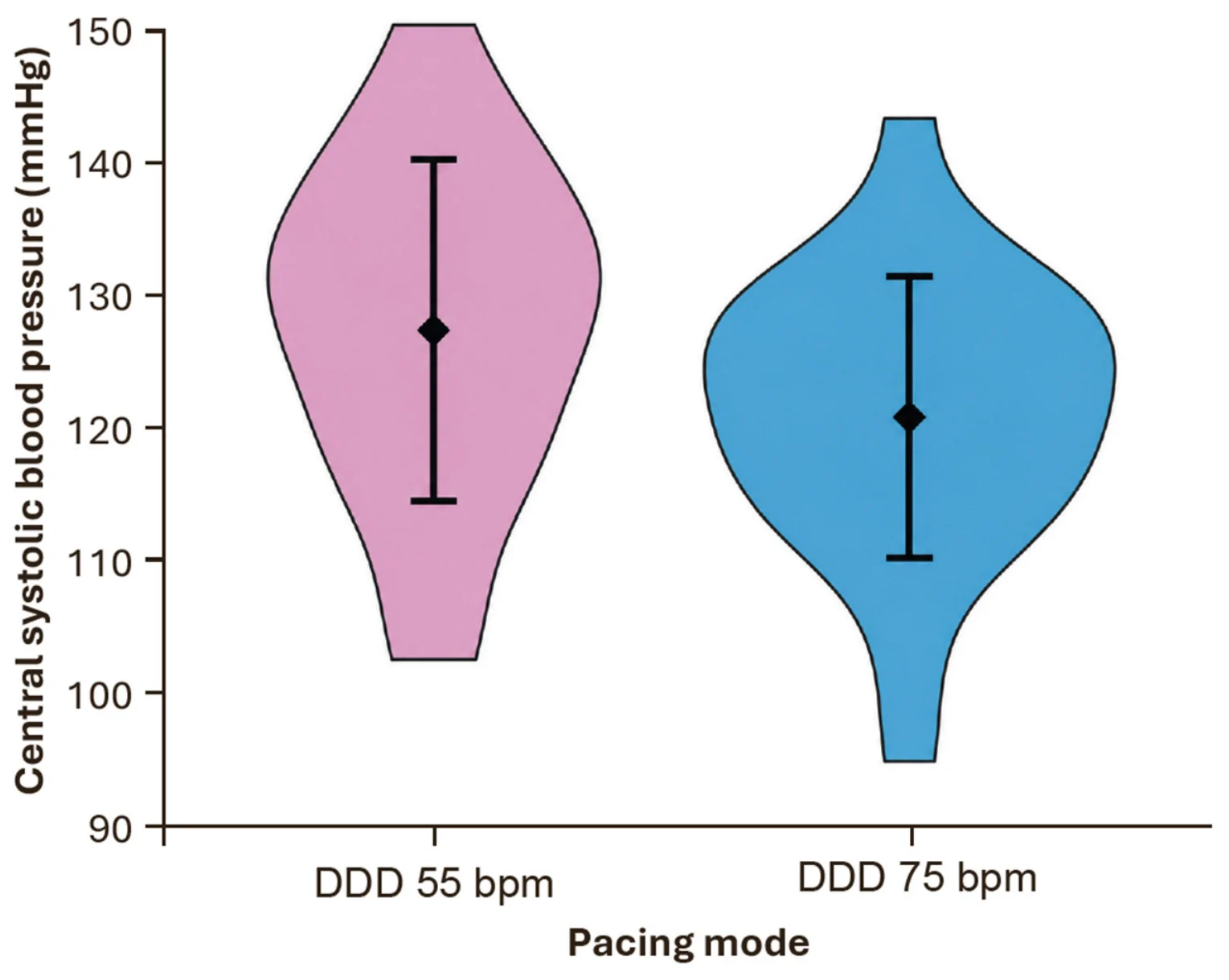
Violin plot showing the distribution of central systolic BP at the lower (DDD 55 bpm; mean 127.8 ± 13.4 mmHg) and higher (DDD 75 bpm; mean 121.3 ± 10.6 mmHg) pacing rate levels (*p* = 0.04).

For the prespecified primary outcome of this exploratory study, central systolic BP was higher at the lower than at the higher pacing rate (mean 127.8 ± 13.4 and 121.3 ± 10.6 mmHg, respectively, β = −6.5 (95% CI: −12.1; −0.8), *p* = 0.04, R^2^ = 43%, Table 2, Figure 2).

After adjustment for peripheral systolic BP, pacing rate level remained significantly associated with central systolic BP in a multivariable linear mixed-effects model (β = −2.2 (95% CI: −3.2; −1.2), *p* < 0.01).

No significant period or sequence (carryover) effects were observed for the primary outcome (*p* = 0.45 and *p* = 0.34, respectively), and the period-adjusted estimate was virtually identical to that of the primary model (central systolic BP: β = −6.7 (95% CI: −12.4; −1.0), *p* = 0.03, Supplementary Tables S1 and S2).

Post hoc sensitivity analyses addressing atrial fibrillation episodes yielded effect estimates of similar magnitude and direction for the primary outcome, although the smaller analytic samples resulted in wider confidence intervals, several of which included zero (Supplementary Table S3).

### 3.4. Secondary Outcomes

Exploratory secondary outcomes are detailed in Table 2. At DDD 55 bpm, peripheral systolic BP was higher than at DDD 75 bpm, but the difference remained statistically nonsignificant (*p* = 0.15). From the lower to the higher pacing rate level, peripheral diastolic BP, central diastolic BP and mean arterial pressure increased (respective *p*-values < 0.01, < 0.01 and 0.03).

From the lower to the higher pacing rate level, central AIx and central AP decreased (*p* = 0.02 and *p* < 0.01, respectively) because of a decrease in P2 pressure and no change in P1 pressure. PP amplification increased from DDD 55 to 75 bpm (*p* < 0.01), driven by a larger decrease in central compared to peripheral PP. Systolic BP amplification increased from the lower to the higher pacing rate level (*p* = 0.02), showing a more pronounced decrease in central compared to peripheral systolic BP with pacing rate increase in this study. P1 height decreased significantly from the lower to the higher pacing rate level (*p* < 0.01).

Cf-PWV increased nonsignificantly from the lower to the higher pacing rate level (*p* = 0.07). No significant associations were found between central systolic BP and baseline cf-PWV (r = 0.05, *p* = 0.84) or central PP and baseline cf-PWV (r = 0.26, *p* = 0.25) after excluding one outlier with an extreme baseline cf-PWV value.

## 4. Discussion

In this exploratory study, we evaluated the long-term effects of two clinically realistic pacing rate strategies on the non-invasively derived central BP profile in adults with SND, a permanent dual-chamber cardiac pacemaker and concomitant medically treated primary arterial hypertension. The primary outcome of the study was that at the lower pacing rate level (DDD 55 bpm), central systolic BP was significantly higher than at the higher pacing rate level (DDD 75 bpm).

### 4.1. Peripheral Blood Pressure Profile

To facilitate interpretation of the primary outcome—central systolic BP—we first address peripheral BP, which is more familiar and provides key determinants of central BP.

At DDD 75 bpm, peripheral diastolic BP and mean arterial pressure were higher than at DDD 55 bpm. This is consistent with the disproportionate shortening of diastole relative to systole at higher HR, which limits the time for arterial pressure to decay between beats and thereby raises end-diastolic arterial pressure [10].

The behaviour of peripheral systolic BP in this study is difficult to interpret given the limited availability of direct markers of left ventricular performance and vascular function. We observed a significant decrease in P1 height, a surrogate of the forward central pressure wave, from the lower to the higher pacing rate level. This finding could be compatible with changes in myocardial contractility due to, for example, pacing-induced sympathoinhibition [20,21], and/or changes in left ventricular volumetric parameters reported in previous pacing studies [22,23,24], although these mechanisms were not directly assessed in our population. Moreover, much of the available evidence on ventricular performance is derived from acute pacing studies and, in part, from HFpEF cohorts, limiting direct extrapolation to the present population. Although hypertension-associated structural remodelling and diastolic abnormalities, which were also present in some participants in our cohort, may represent a substrate for HFpEF, such findings do not in themselves establish the haemodynamic phenotype of clinically overt HFpEF [25]. The underlying mechanism of the decrease in P1 height therefore remains uncertain. Although cardiac output was not directly measured, the observed haemodynamic pattern does not suggest a substantial increase in cardiac output with pacing rate elevation. Combined with the modest increase in diastolic pressure from DDD 55 to 75 bpm, the lower P1 height may have contributed to the observed numerical decrease in peripheral systolic BP, although this did not reach statistical significance. Peripheral vascular adjustments and Frank–Starling effects may also have contributed to peripheral systolic BP behaviour [26].

Expressed more simply, a smaller rise in peripheral diastolic BP than the fall in peripheral PP with pacing rate elevation produced a slight net peripheral systolic BP decrease, since systolic BP = diastolic BP + PP.

### 4.2. Central Blood Pressure Profile

Since peripheral pressures generally account for much of the variability in central pressures [27,28,29], it is unsurprising that central systolic BP and central PP declined with pacing rate elevation in the study. The magnitude of the central systolic BP and central PP reduction, however, exceeded that of the peripheral changes, as reflected by the significant rise in systolic BP and PP amplifications from DDD 55 to 75 bpm. Since pacing rate level remained significantly associated with central systolic BP after adjusting for peripheral systolic BP in a multivariable linear mixed-effects model, we explain this phenomenon with shortened ejection duration at the higher pacing rate level, that shifts the timing of the reflected wave towards diastole, thereby reducing central AP and central AIx, and facilitating central systolic BP to decrease more than peripheral systolic BP [15,30].

The observed reduction in central systolic BP from DDD 55 to 75 bpm may also reflect a lower forward wave amplitude, as suggested by lower P1 height at the higher pacing rate level in our study. As central systolic BP represents the combined effect of forward and reflected waves, a smaller forward component diminishes the level upon which reflections act.

Regarding the effect of aortic stiffness on central systolic BP, a slightly higher cf-PWV at the higher pacing rate could theoretically have reduced the magnitude of central systolic BP decrease from the lower to the higher pacing rate level. In stiff arteries, as typically seen in older individuals, higher PWV leads to larger reflected wave amplitudes and an earlier return of these waves to the aorta [10,31,32,33], thus augmenting central systolic BP.

We previously studied the role of paced HR on central BP indices in SND patients with a permanent cardiac pacemaker across the pacing rate range from 40 to 90 bpm in an acute setting [14]. Although an acute interaction between HR and BP generally differs from the long-term response, the effect of HR on the relationship between peripheral and central pressures appears consistent in both settings: higher HR enhances pressure amplification because the aortic–brachial pressure differences are determined primarily by the physical properties of the arterial transfer function [10]. Accordingly, we observed similar overall effects of paced HR on systolic BP and PP amplifications in the long-term study as in the acute setting.

### 4.3. Clinical Implications

Although caution is warranted when drawing clinical conclusions from this exploratory study, it is reasonable to consider the potential clinical implications of our findings.

In a meta-analysis of 48,200 participants with and without hypertension, a 10 mmHg higher central systolic BP was associated with a 13% higher risk of composite cardiovascular events after adjustment for peripheral systolic BP [34]. In the CAFE substudy of ASCOT, the treatment groups had similar peripheral systolic BP but differed by approximately 4.3 mmHg in central systolic BP, supporting the concept that relatively modest differences in central pressure may reflect haemodynamic differences not captured by peripheral BP alone [35]. In this context, the primary finding of the present study—a nearly 7 mmHg lower achieved central systolic BP at the higher versus the lower pacing rate—may have potential clinical relevance. However, after adjustment for peripheral systolic BP, the residual association between pacing rate and central systolic BP was smaller (β = −2.2 mmHg). Thus, the observed difference in central systolic BP appears to reflect both changes in peripheral pressure and an additional central haemodynamic component.

Other surrogate markers of clinical outcomes assessed in our study—peripheral systolic BP [36] and peripheral PP [37], as well as central AP and central PP [38]—likewise indicate a potentially more favourable haemodynamic profile during higher-rate pacing compared to slightly bradycardic pacing in SND patients with hypertension. Finally, the greater systolic BP and PP amplification observed at the higher pacing rate suggests that, at a given peripheral systolic BP (or peripheral PP), central systolic BP (or central PP) may be slightly lower at higher pacing rates [10], which may be relevant to central pressure exposure of target organs.

It lies beyond the scope of our study to determine whether pacing rate in patients with SND and hypertension should be tailored according to (central) BP. Nonetheless, the question is timely and potentially clinically relevant in light of increasing interest in device-based hypertension control strategies in pacemaker recipients [13]. Pacemaker-based atrioventricular interval modulation (AVIM) therapy, which received a Breakthrough Device designation in 2025 [39], lowers peripheral BP by reducing left ventricular filling pressures and total peripheral resistance, while simultaneously modulating autonomic nervous system activity [40]. Similar neuromodulatory effects may have contributed to BP changes in our study. Given that up to 70% of pacemaker recipients have hypertension [41], the relationship between pacemaker programming and BP warrants further investigation.

### 4.4. Limitations

This study has several limitations. First, the target enrolment of 58 participants was not achieved, primarily due to the COVID-19 pandemic, resulting in substantially lower statistical power than originally planned. The small sample size limits the precision of the effect estimate and increases uncertainty regarding its magnitude, which was also reflected in the wider confidence intervals observed in post hoc sensitivity analyses excluding participants or measurements potentially affected by atrial fibrillation episodes. Second, the predominance of older, female participants and the high prevalence of beta-blocker use in our cohort limit the generalisability of the findings. Third, data on left ventricular function, vascular resistance, autonomic nervous system activity and wave separation were not collected, which limited interpretation of the findings. Fourth, because the participants had moderately well-controlled hypertension at baseline, the potential for further changes in BP was inherently limited, which may have influenced the observed magnitude of the effect. Extrapolation of these findings to patients with drug-resistant hypertension should thus be made with caution. Fifth, the exclusion criteria were extensive. However, this primarily reflected the study’s mechanistic (physiological) focus rather than a clinical one.

## 5. Conclusions

Long-term pacing at a moderately accelerated physiological rate may be associated with lower central systolic BP compared with pacing at a slightly bradycardic rate, in older permanently paced SND patients with hypertension. For the same level of peripheral systolic BP or peripheral PP, central systolic BP or central PP may be slightly lower at higher paced rates. Further studies are needed to determine the clinical relevance of these haemodynamic findings.

## Figures and Tables

**Table 1:** Baseline characteristics of the subjects.

Parameter	Values
**Demographic characteristics**	
Age, years	76 (73–78)
Male sex, n (%)	6 (27)
Weight, kg	80.5 ± 13.9
Body mass index, kg/m^2^	29.9 ± 4.5
Never smoker, n (%)	18 (82)
**Disease characteristics, n (%)**	
Dyslipidaemia	18 (82)
Atrial fibrillation or flutter	16 (73)
Obesity (BMI ≥ 30 kg/m^2^)	9 (41)
**Pacemaker characteristics**	
Atrial pacing, %	96 (94–98)
Ventricular pacing < 1%, n (%)	13 (59)
Auto-mode switch from previous interrogation	0 (0)
**Cardiovascular medication, n (%)**	
Number of antihypertensives	2.5 ± 0.8
ACE inhibitors/ARBs	21 (95)
Calcium channel blockers	8 (36)
Diuretics	7 (32)
Beta-blockers	18 (82)
Statins	14 (64)
Antiplatelets	1 (5)
Anticoagulants	14 (64)
**Blood analyses**	
Glucose, mmol/L	6 (5–6)
Total cholesterol, mmol/L	5.4 ± 1.1
LDL cholesterol, mmol/L	3 (3–4)
Thyroid stimulating hormone, µU/L	2 (2–3)
Haemoglobin, g/L	133.5 ± 9.6
eGFR, mL/min/1.73 m^2^	71 (55–82)
NT-proBNP, pg/mL	196 (134–525)
**Haemodynamic characteristics**	
Peripheral systolic BP, mmHg	130.3 ± 11.7
Peripheral diastolic BP, mmHg	80.6 ± 6.7
Central systolic BP, mmHg	125.0 ± 13.4
Central diastolic BP, mmHg	80.1 ± 6.0
Carotid–femoral pulse wave velocity, m/s	9.7 ± 1.9
Augmentation index, %	33.4 ± 8.6
Heart rate, bpm	64.7 ± 1.8
**Echocardiographic parameters**	
Left ventricular end-diastolic diameter, mm	46.8 ± 6.0
Interventricular septal wall thickness, mm	10 (9–10)
Posterior wall thickness, mm	9 (8–10)
Relative wall thickness	0.38 (0.35–0.41)
Left ventricular mass index, g/m^2^	82.9 ± 21.6
Left ventricular ejection fraction, %	64.6 ± 6.2
Left atrial end-systolic volume index, ml/m^2^	34.9 ± 11.0
Average mitral E/e’	11.8 ± 5.5
Pulmonary artery systolic pressure, mmHg	30.3 ± 6.6

Values are presented as mean ± SD, median (IQR) or count (%). BMI indicates body mass index; ACE, angiotensin-converting enzyme; ARB, angiotensin receptor blocker; LDL, low-density lipoprotein; eGFR, estimated glomerular filtration rate; NT-proBNP, N-terminal pro-brain natriuretic peptide; and BP, blood pressure.

**Table 2:** Haemodynamic indices at lower (DDD 55 bpm) and higher (DDD 75 bpm) pacing rate levels and their association with pacing level assessed using linear mixed-effects models.

Parameter	DDD 55 bpm	DDD 75 bpm	β (95% CI)	R^2^ (%)	*p*-Value
Heart rate, bpm	54.5 ± 1.5	74.8 ± 1.4			
**Primary outcome**					
Central systolic BP, mmHg	127.8 ± 13.4	121.3 ± 10.6	−6.5 (−12.1; −0.8)	43	0.04
**Secondary outcomes**					
Peripheral systolic BP, mmHg	136.3 ± 15.1	131.4 ± 12.4	−4.9 (−11.3; 1.5)	42	0.15
Peripheral diastolic BP, mmHg	74.2 ± 6.2	81.0 ± 5.6	6.8 (3.5; 10.1)	36	<0.01
Peripheral pulse pressure, mmHg	62.0 ± 13.8	50.4 ± 10.3	−11.6 (−15.8; −7.4)	73	<0.01
Central diastolic BP, mmHg	75.2 ± 6.0	81.3 ± 5.5	6.2 (3; 9.3)	35	<0.01
Central pulse pressure, mmHg	52.6 ± 12.1	40.0 ± 8.1	−12.7 (−16.3; −9.0)	74	<0.01
Mean arterial pressure, mmHg	93.8 ± 7.4	98.5 ± 7.2	4.7 (0.8; 8.6)	28	0.03
Pulse pressure amplification (peripheral/central PP)	1.2 ± 0.1	1.3 ± 0.1	0.1 (0.1; 0.1)	65	<0.01
Systolic BP amplification (peripheral-central systolic BP), mmHg	8.5 ± 3.2	10.1 ± 2.9	1.6 (0.3; 2.8)	56	0.02
P1, mmHg	107.1 ± 11.0	107.9 ± 8.7	0.8 (−4.3; 5.9)	26	0.76
P1 height (P1—minimum pressure of the central waveform), mmHg	37.8 ± 8.6	30.9 ± 6.4	−7.0 (−9.5; −4.5)	80	<0.01
P2, mmHg	127.8 ± 13.4	121.3 ± 10.6	−6.5 (−12.1; −0.8)	43	0.04
Central augmented pressure, mmHg	20.7 ± 7.8	13.4 ± 5.0	−7.3 (−9.9; −4.6)	66	<0.01
Central augmentation index, %	38.7 ± 10.1	33.1 ± 9.1	−5.6 (−9.9; −1.4)	50	0.02
Ejection duration, msec	349.1 ± 28.6	310.9 ± 22.0	−39.8 (−47.8; −31.7)	86	<0.01
Carotid–femoral pulse wave velocity, m/s	9.4 ± 1.6	10.1 ± 1.9	0.7 (0; 1.3)	62	0.07

Values are presented as mean ± SD. DDD indicates dual-chamber paced, dual-chamber sensed, dual response mode; BP, blood pressure; PP, pulse pressure; P1, pressure at the first systolic peak of the central pressure waveform; and P2, pressure at the second systolic peak of the central pressure waveform.

## Data Availability

The datasets used and/or analysed during the current study are available from the corresponding author on reasonable request.

## Supplementary Materials

The following supporting information can be downloaded at: https://www.mdpi.com/article/10.3390/medicina62102032/s1, Supplementary Materials: Exclusion criteria of the study; Sample size calculation; Supplementary Table S1. Assessment of period and sequence (carryover) effects for the primary and secondary outcomes using linear mixed-effects models; Supplementary Table S2. Haemodynamic indices by treatment sequence and study period; Supplementary Table S3. Sensitivity analyses for central and peripheral systolic blood pressure excluding participants or measurements affected by atrial fibrillation episodes; Supplementary Figure S1. CONSORT 2025 flow diagram of the study.

## Abbreviations

The following abbreviations are used in this manuscript:

ACE	Angiotensin-converting enzyme
AIx	Augmentation index
AP	Augmented pressure
ARB	Angiotensin receptor blocker
AVIM	Atrioventricular interval modulation
BMI	Body mass index
BP	Blood pressure
cf-PWV	Carotid–femoral pulse wave velocity
CI	Confidence interval
DDD	Dual-chamber paced, dual-chamber sensed, dual response mode
ECG	Electrocardiogram
eGFR	Estimated glomerular filtration rate
HFpEF	Heart failure with preserved ejection fraction
HR	Heart rate
IQR	Interquartile range
LDL	Low-density lipoprotein
NT-proBNP	N-terminal pro-brain natriuretic peptide
PP	Pulse pressure
PWA	Pulse wave analysis
PWV	Pulse wave velocity
SD	Standard deviation
SND	Sinus node dysfunction
