# Supplementary material for: Long-Term Effects of Paced Heart Rate on Central Blood Pressure in Patients with Sinus Node Dysfunction and Hypertension: A Randomised Crossover Study
Source: Medicina (Kaunas). 2026 Oct 20;62(10):2032. doi: 10.3390/medicina62102032 (PMC13706900; doi:10.3390/medicina62102032)

Long-term implications of paced heart rate on central blood pressure in adult patients with sinus node dysfunction, a permanent pacemaker and hypertension: a randomised crossover study

Supplementary Materials

Exclusion criteria of the study

The exclusion criteria were as follows: all inclusion criteria not met; average seated office peripheral systolic BP ≥ 140 mmHg and/or diastolic BP ≥ 90 mmHg; inter-arm difference in peripheral systolic BP ≥ 15 mmHg; atrial pacing < 80% or ventricular pacing > 3% since last pacemaker interrogation; unpaced QRS > 130 ms or QTc > 500 ms on 12-lead ECG; central hemodynamic measurements cannot be reliably performed; irregular HR on 12-lead ECG; frequent episodes of atrial high rate (clinically or automatic mode switching ≥ 3%); implantable cardioverter defibrillator or cardiac resynchronization therapy pacemaker; treatment with digoxin, ivabradine or Vaughan-Williams class Ic, III or IV antiarrhythmic drugs; treatment with > 3 antihypertensive medications; treatment with an inhaled beta-adrenergic agonist; secondary or resistant hypertension; orthostatic hypotension; history of acute coronary syndrome or coronary artery bypass grafting; stable angina pectoris; heart failure with reduced ejection fraction; heart failure with preserved ejection fraction (NYHA > II); history of a cerebrovascular event; degenerative central nervous system disease; body mass index ≥ 35 kg/m2; type I diabetes mellitus; type II diabetes mellitus with cardiovascular complications or on insulin therapy; other significant endocrine disorder; chronic kidney disease with an estimated glomerular filtration rate of < 30 ml/min/m2; symptomatic peripheral artery disease; clinically relevant heart valve disease; congenital heart disease; cancer treatment within 5 years; systemic connective tissue disease; anemia; severe chronic respiratory or liver disease; absence of Estonian Health Insurance Fund coverage; inability to comply with the study protocol; withdrawal of consent or loss of contact during the study; alcohol abuse; and pregnancy or breastfeeding.

Sample size calculation

Assuming an intra-individual difference in central systolic BP between interventions of at least 4 mmHg (SD 9.6 mmHg), a sample size of 48 participants was required to achieve 80% power at a two-sided significance level of 0.05. Accounting for an expected dropout rate of 20%, the required total sample size was 58 participants.

Supplementary Table S1. Assessment of period and sequence (carryover) effects for the primary and secondary outcomes using linear mixed-effects models.

Parameter	n	Primary model		Period-adjusted model				Sequence (carryover)
		β (95% CI)	P-value	Pacing rate β (95% CI)	P-value	Period β (95% CI)	P-value	P-value
Primary outcome								
Central systolic BP, mm Hg	22	−6.5 (−12.1; −0.8)	0.035	−6.7 (−12.4; −1.0)	0.033	−2.3 (−8.0; 3.5)	0.447	0.342
Secondary outcomes								
Peripheral systolic BP, mm Hg	22	−4.9 (−11.3; 1.5)	0.148	−5.1 (−11.6; 1.4)	0.138	−2.4 (−8.9; 4.1)	0.483	0.285
Peripheral diastolic BP, mm Hg	22	6.8 (3.5; 10.1)	<0.001	6.7 (3.4; 10.0)	<0.001	−0.9 (−4.2; 2.5)	0.616	0.507
Peripheral pulse pressure, mm Hg	22	−11.6 (−15.8; −7.4)	<0.001	−11.7 (−16.0; −7.4)	<0.001	−1.2 (−5.5; 3.1)	0.583	0.450
Central diastolic BP, mm Hg	22	6.2 (3.0; 9.3)	<0.001	6.1 (2.9; 9.3)	0.001	−0.7 (−3.9; 2.6)	0.688	0.415
Central pulse pressure, mm Hg	22	−12.7 (−16.3; −9.0)	<0.001	−12.8 (−16.5; −9.1)	<0.001	−1.6 (−5.3; 2.1)	0.411	0.547
Mean arterial pressure, mm Hg	22	4.7 (0.8; 8.6)	0.027	4.6 (0.6; 8.6)	0.034	−1.2 (−5.2; 2.8)	0.561	0.305
Pulse pressure amplification	22	0.08 (0.05; 0.11)	<0.001	0.08 (0.06; 0.11)	<0.001	0.02 (−0.01; 0.04)	0.167	0.561
Systolic BP amplification, mm Hg	22	1.6 (0.3; 2.8)	0.023	1.6 (0.3; 2.8)	0.028	−0.1 (−1.4; 1.2)	0.871	0.345
P1, mm Hg	22	0.8 (−4.3; 5.9)	0.764	0.6 (−4.6; 5.8)	0.828	−2.4 (−7.6; 2.8)	0.378	0.183
P1 height, mm Hg	20	−7.0 (−9.5; −4.5)	<0.001	−7.1 (−9.7; −4.5)	<0.001	−0.6 (−3.2; 2.0)	0.634	0.656
P2, mm Hg	22	−6.5 (−12.1; −0.8)	0.035	−6.7 (−12.4; −1.0)	0.033	−2.3 (−8.0; 3.5)	0.447	0.342
Central augmented pressure, mm Hg	22	−7.3 (−9.9; −4.6)	<0.001	−7.3 (−10.0; −4.6)	<0.001	0.1 (−2.6; 2.8)	0.936	0.889
Central augmentation index, %	22	−5.6 (−9.9; −1.4)	0.017	−5.5 (−9.8; −1.2)	0.022	1.3 (−3.0; 5.6)	0.565	0.580
Ejection duration, msec	20	−39.8 (−47.8; −31.7)	<0.001	−40.8 (−48.7; −32.9)	<0.001	−6.0 (−13.9; 1.9)	0.159	0.379
Carotid-femoral pulse wave velocity, m/s	22	0.7 (0.0; 1.3)	0.068	0.6 (−0.1; 1.3)	0.091	−0.3 (−1.0; 0.3)	0.329	0.959

Primary model: outcome ~ pacing rate + age + (1 | participant), as reported in Table 2. Period-adjusted model: period (second vs first intervention period) added as a fixed effect. Sequence P-value: from a model additionally including treatment sequence (DDD 55 → 75 vs DDD 75 → 55); in a two-treatment, two-period crossover design, the sequence effect is aliased with the carryover (treatment-by-period interaction) effect. β indicates the estimated difference at DDD 75 vs DDD 55 bpm (pacing rate) or in the second vs the first period (period). P-values were obtained with F-tests using the Kenward–Roger approximation for denominator degrees of freedom; 95% CIs are Wald intervals. BP indicates blood pressure; P1, pressure at the first systolic peak; P2, pressure at the second systolic peak.

Supplementary Table S2. Haemodynamic indices by treatment sequence and study period.

Parameter	Sequence DDD 55 → 75 (n = 10)			Sequence DDD 75 → 55 (n = 12)		
	Period 1 (DDD 55)	Period 2 (DDD 75)	Difference 75−55 (95% CI)	Period 1 (DDD 75)	Period 2 (DDD 55)	Difference 75−55 (95% CI)
Heart rate, bpm	54.3 ± 0.6	75.0 ± 0.2	20.8 (20.3; 21.2)	74.7 ± 1.9	54.6 ± 2.0	20.0 (18.4; 21.7)
Primary outcome						
Central systolic BP, mm Hg	131.2 ± 12.9	122.3 ± 4.8	−8.9 (−17.5; −0.4)	120.5 ± 13.9	124.9 ± 13.6	−4.4 (−13.9; 5.0)
Secondary outcomes						
Peripheral systolic BP, mm Hg	140.4 ± 14.7	132.9 ± 6.6	−7.5 (−18.1; 3.1)	130.1 ± 16.0	132.9 ± 15.2	−2.8 (−12.9; 7.4)
Peripheral diastolic BP, mm Hg	75.4 ± 8.0	81.2 ± 4.5	5.8 (0.6; 11.1)	80.8 ± 6.6	73.2 ± 4.3	7.6 (2.3; 12.9)
Peripheral pulse pressure, mm Hg	64.5 ± 10.9	51.6 ± 7.3	−12.9 (−19.4; −6.4)	49.3 ± 12.5	59.8 ± 15.9	−10.5 (−17.5; −3.4)
Central diastolic BP, mm Hg	76.4 ± 7.8	81.8 ± 4.4	5.5 (0.3; 10.6)	80.9 ± 6.5	74.1 ± 4.1	6.8 (1.6; 11.9)
Central pulse pressure, mm Hg	54.9 ± 9.0	40.5 ± 5.7	−14.4 (−18.9; −9.9)	39.6 ± 9.9	50.8 ± 14.4	−11.2 (−17.9; −4.5)
Mean arterial pressure, mm Hg	95.8 ± 9.0	99.2 ± 3.5	3.4 (−2.6; 9.4)	97.8 ± 9.4	92.0 ± 5.6	5.8 (−0.7; 12.3)
Pulse pressure amplification	1.18 ± 0.07	1.28 ± 0.06	0.10 (0.06; 0.14)	1.25 ± 0.05	1.18 ± 0.06	0.06 (0.02; 0.10)
Systolic BP amplification, mm Hg	9.2 ± 3.7	10.6 ± 2.7	1.4 (−1.3; 4.2)	9.6 ± 3.0	8.0 ± 2.8	1.7 (0.2; 3.2)
P1, mm Hg	110.8 ± 12.8	109.0 ± 6.0	−1.8 (−11.3; 7.7)	107.0 ± 10.6	104.0 ± 8.7	3.0 (−4.3; 10.3)
P1 height, mm Hg	39.3 ± 4.6	31.6 ± 5.2	−8.0 (−11.0; −5.0)	30.3 ± 7.5	36.9 ± 10.3	−6.0 (−10.6; −1.5)
P2, mm Hg	131.2 ± 12.9	122.3 ± 4.8	−8.9 (−17.5; −0.4)	120.5 ± 13.9	124.9 ± 13.6	−4.4 (−13.9; 5.0)
Central augmented pressure, mm Hg	20.4 ± 6.7	13.3 ± 4.5	−7.2 (−10.7; −3.6)	13.5 ± 5.6	20.9 ± 9.0	−7.4 (−12.1; −2.6)
Central augmentation index, %	36.9 ± 10.2	32.6 ± 10.4	−4.2 (−11.1; 2.7)	33.4 ± 8.2	40.2 ± 10.2	−6.8 (−13.7; 0.1)
Ejection duration, msec	360.0 ± 29.3	312.8 ± 17.1	−47.1 (−62.2; −32.0)	309.4 ± 26.1	342.8 ± 27.4	−34.6 (−46.4; −22.8)
Carotid-femoral pulse wave velocity, m/s	9.6 ± 2.0	10.0 ± 1.8	0.2 (−0.7; 1.1)	10.2 ± 2.1	9.2 ± 1.2	1.0 (−0.2; 2.1)

Values are presented as mean ± SD measured at the end of each eight-week intervention period. Within-sequence differences (DDD 75 − DDD 55 bpm) are presented as mean (95% CI) of paired differences. Abbreviations as in Supplementary Table S1.

Supplementary Table S3. Sensitivity analyses for central and peripheral systolic blood pressure excluding participants or measurements affected by atrial fibrillation episodes.

Analysis	Participants, n	Measurements, n	Primary model		Period-adjusted model	
			β (95% CI)	P-value	β (95% CI)	P-value
Central systolic BP, mm Hg						
All participants (main analysis)	22	44	−6.5 (−12.1; −0.8)	0.035	−6.7 (−12.4; −1.0)	0.033
Excluding participants with AF episodes	19	38	−6.1 (−12.6; 0.4)	0.083	−6.7 (−13.5; 0.2)	0.073
Excluding AF-affected measurements only	22	41	−6.2 (−12.4; 0.0)	0.066	−6.6 (−13.0; −0.2)	0.058
Excluding participant A (AF during DDD 75 bpm)	21	42	−6.0 (−11.9; −0.2)	0.057	−6.3 (−12.3; −0.3)	0.053
Excluding participant B (AF during washout after DDD 55 bpm)	21	42	−6.5 (−12.4; −0.5)	0.045	−6.8 (−12.8; −0.7)	0.040
Excluding participant C (AF during DDD 55 bpm)	21	42	−6.6 (−12.5; −0.7)	0.042	−6.9 (−13.0; −0.9)	0.036
Peripheral systolic BP, mm Hg						
All participants (main analysis)	22	44	−4.9 (−11.3; 1.5)	0.148	−5.1 (−11.6; 1.4)	0.138
Excluding participants with AF episodes	19	38	−4.4 (−11.8; 2.9)	0.249	−5.1 (−12.8; 2.7)	0.218
Excluding AF-affected measurements only	22	41	−4.5 (−11.5; 2.4)	0.220	−5.0 (−12.2; 2.3)	0.196
Excluding participant A (AF during DDD 75 bpm)	21	42	−4.3 (−10.9; 2.3)	0.214	−4.6 (−11.4; 2.2)	0.201
Excluding participant B (AF during washout after DDD 55 bpm)	21	42	−4.9 (−11.6; 1.8)	0.168	−5.3 (−12.1; 1.6)	0.150
Excluding participant C (AF during DDD 55 bpm)	21	42	−5.1 (−11.8; 1.6)	0.154	−5.5 (−12.3; 1.4)	0.135

β indicates the estimated difference between DDD 75 and DDD 55 bpm. Primary model: outcome ~ pacing rate + age + (1 | participant), as reported in Table 2. Period-adjusted model: period (second vs first intervention period) added as a fixed effect. Three participants experienced four AF episodes requiring rhythm restoration: participant A during DDD 75 bpm, participant B during the washout following DDD 55 bpm, and participant C (two episodes) during DDD 55 bpm. AF-affected measurements were defined as the end-of-period measurement following the AF episode (DDD 75 bpm for participants A and B; DDD 55 bpm for participant C). P-values were obtained with F-tests using the Kenward–Roger approximation for denominator degrees of freedom; 95% CIs are Wald intervals. AF indicates atrial fibrillation; BP, blood pressure.

Supplementary Figure S1: CONSORT 2025 flow diagram of the study.

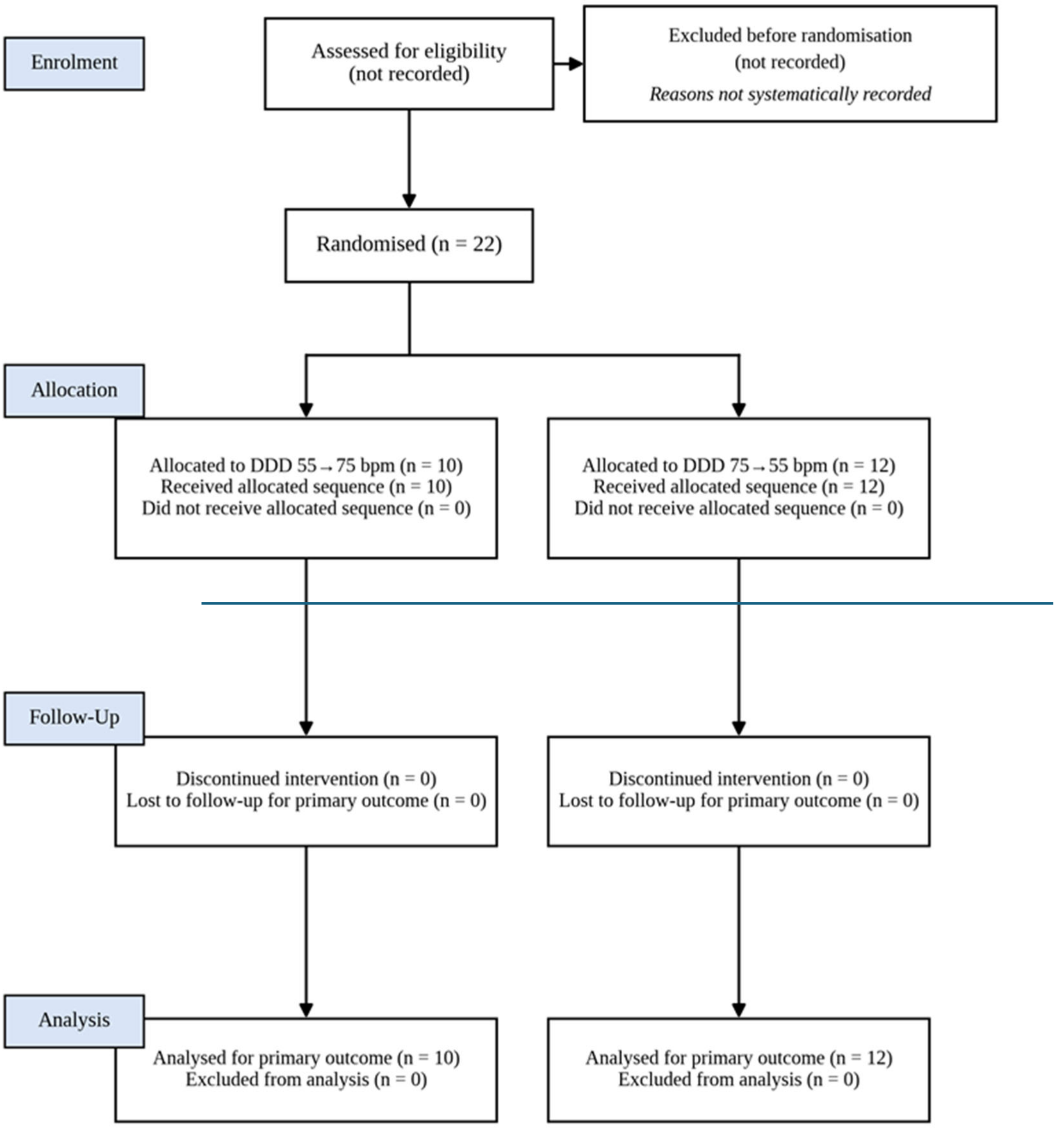

Supplement: Supplementary file 1 [file medicina-62-02032-s001.zip › medicina-4556357-supplementary.pdf]
